# CeCaFDB: a curated database for the documentation, visualization and comparative analysis of central carbon metabolic flux distributions explored by 13C-fluxomics

**DOI:** 10.1093/nar/gku1137

**Published:** 2014-11-11

**Authors:** Zhengdong Zhang, Tie Shen, Bin Rui, Wenwei Zhou, Xiangfei Zhou, Chuanyu Shang, Chenwei Xin, Xiaoguang Liu, Gang Li, Jiansi Jiang, Chao Li, Ruiyuan Li, Mengshu Han, Shanping You, Guojun Yu, Yin Yi, Han Wen, Zhijie Liu, Xiaoyao Xie

**Affiliations:** 1 College of Computer Science and Technology, Guizhou University, Guiyang, Guizhou 550025, P.R. China; 2 Key Laboratory of Information and Computing Science Guizhou Province, Guizhou Normal University, Guiyang, Guizhou 563000, P. R. China; 3 School of Life Sciences, Anhui Agricultural University, Hefei, Anhui 230026, P. R. China; 4 School of Life Sciences, Guizhou Normal University, Guiyang, Guizhou 563000, P. R. China

## Abstract

The Central Carbon Metabolic Flux Database (CeCaFDB, available at http://www.cecafdb.org) is a manually curated, multipurpose and open-access database for the documentation, visualization and comparative analysis of the quantitative flux results of central carbon metabolism among microbes and animal cells. It encompasses records for more than 500 flux distributions among 36 organisms and includes information regarding the genotype, culture medium, growth conditions and other specific information gathered from hundreds of journal articles. In addition to its comprehensive literature-derived data, the CeCaFDB supports a common text search function among the data and interactive visualization of the curated flux distributions with compartmentation information based on the Cytoscape Web API, which facilitates data interpretation. The CeCaFDB offers four modules to calculate a similarity score or to perform an alignment between the flux distributions. One of the modules was built using an inter programming algorithm for flux distribution alignment that was specifically designed for this study. Based on these modules, the CeCaFDB also supports an extensive flux distribution comparison function among the curated data. The CeCaFDB is strenuously designed to address the broad demands of biochemists, metabolic engineers, systems biologists and members of the -omics community.

## INTRODUCTION

Upon entering the post-genome period, the emergence of modern technology has driven a huge wave of -omics research, such as transcriptomics, proteomics, metabolomics, 13C-fluxomics and so on (1,2). As massive amount of data has been accumulated through projects using various -omics, well-known databases have emerged in large numbers for the distribution and comparative analysis of these data (3,4). A number of transcriptome databases have arisen for general and specific purposes, such as the general-purpose Microbial Transcriptome Database and HBT for Human transcriptome (5–7). In addition, there has been a tremendous increase in the number of databases for metabolomics and metabolic pathway data. MetaboLights was constructed for documentation of metabolite structures, their reference spectra and experimental data from metabolic experiments (8). The Human Metabolome Database was built as a database containing detailed information regarding small molecule metabolites found in the human body (9). The famous MetaCyc database provides comprehensive and freely accessible resource of metabolic pathways and enzymes (10). The goal of the BiGG database is to address the need for access to high-quality curated metabolic models and reconstructions among the systems biology community (11).

Among these -omics, 13C-fluxomics is a new area that involves experimentally quantifying the rates of metabolic reactions within the central carbon metabolism through ^13^C metabolic fluxomics (12–15). This method enables the resolution of paralleled and reversible reactions (14). Generally, fluxomics delivers a flux estimate describing the metabolic state of a cell at a given time (16). Because metabolic fluxes are the ultimate identifier of a cell's functional state, the result represents the physiological counterpart of its sibling-omics and is supposed to serve as the critical link between genes, proteins, metabolites and the observable phenotype (17). The incorporation of fluxomics as a basal part of the cellular system allows for a deeper understanding of the cellular physiological properties and provides the most comprehensive integration of the different -omics levels (18,19). Recently, 13C-fluxomics has been employed to discriminate between different physiological states, to investigate metabolic responses to genetic manipulations and environmental stress and to monitor the presence of certain metabolic pathways (20–23).

In parallel with other -omics, 13C-fluxomics has documented large amounts of information regarding the flux distribution of numerous organisms (24). Comparative analysis and theoretical simulation of these results prove to be an extraordinarily powerful method for exploring the hidden meanings among numerous fluxomics results (25–28). Comparisons of experimental flux profiling results with those predicated by flux balance analysis have been implemented for normal melanocytes and melanoma cell lines under normoxic and hypoxic conditions, providing a basis for targeting metabolism for the therapeutic benefit of melanoma (26). By comparing the metabolic fluxes of a large number of deletion mutants of *B. subtilis*, Blank *et al*. found that some mutants grew faster than the corresponding wild strain. This result suggests that bacteria may have a regulatory mechanism that ensures that the metabolic network does not function according to maximized biomass production (29). On the basis of a comparative study of flux measurements from nine bacteria and multiobjective optimization theory, Schuetz *et al*. showed that flux states evolve obeying the trade-off between two principles: optimality at one given condition and minimal adjustment between conditions (30). Furthermore, a comparison between the ‘dry’ flux results and ‘wet’ flux results has obtained increasing universality with the progressive demand for simulation in metabolic systems biology (31,32).

However, a large fraction of the fluxomics results has been reported in non-standard graphical form (33). Each of the related metabolic networks was unique and incompatible with each other due to the varied definition of the lumped reactions (34). Furthermore, a common measure is expected for calculating similarities or distances between different flux distributions. As a result, unlike other -omes, there is still future potential for a public and user-friendly online tool for the documentation, visualization and comparative analysis of quantitative flux results. To this end, we present the Central Carbon Metabolic Flux Database (CeCaFDB, http://www.cecafdb.org/), which, to the best of our knowledge, is the first professionally designed online tool for fluxomics data regarding the central carbon metabolic systems of microbes and animal cells as a platform for data distribution, visualization and alignment in the systems biology community.

The CeCaFDB encompasses 581 cases of flux distribution and provides a Cytoscape Web-based (35) interactive visualization service for the flux data stored in the database or submitted by individual users. Furthermore, the CeCaFDB utilizes three different algorithms, including a vector-based method, a stoichiometry-based method and a topology-based method to compare/align different flux distributions, supporting a comprehensive and flexible solution for comparative flux analysis. Together with the curated flux data, a comparison service is capable of performing high-throughput correlation analysis and of evaluating the phylogenetic relationships of currently available flux distributions. We hope that the CeCaFDB will benefit from all of the research related to fluxomics and will grow into an influential functional database for fluxomics and systems biology.

## DATABASE DESCRIPTION

### Data Source

**Figure 1.**
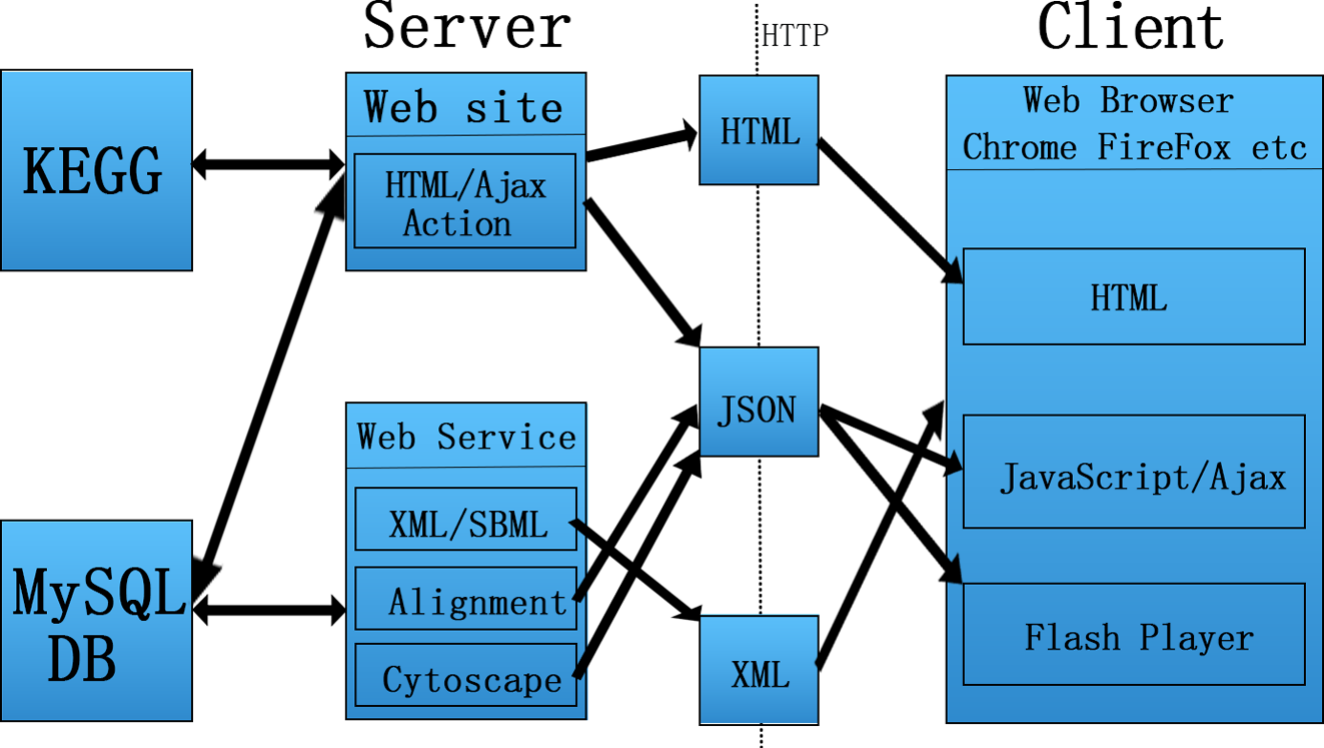
Representation of the system architecture for the CeCaFDB.

The PubMed reference database was queried using various combinations of keywords, such as ‘13C’, ‘metabolic’, ‘flux’ and ‘analysis’, returning approximately 1000 literature results from 1995 to 2013. Seventy percents of them were automatically excluded due to the absence of quantitative flux distribution information. Then a further 10% were measured using a non-13C method and were also omitted. Ultimately, a total of 118 references were collected as a preliminary source for our database. The diversity of the reactions and network definition, the quantity of experimental data and the required genetic and cultivation knowledge made the assembly of the CeCaFDB both difficult and time consuming. The lumped reactions in these studies were broken down into their original forms, as in the KEGG Reaction Database (36), and the flux value was mapped to its precisely corresponding reaction. Currently, the database encompasses 581 cases of flux distributions from 36 organisms. The fluxomics result is displayed in table format with an interactive graphic representation based on the Cytoscape Web platform. The technical architecture of CeCaFDB was shown in Figure 1. The basal statistics are displayed in Table 1.

**Table 1.** The basal statistics of all collected flux distributions

Species	Reference	Condition^a^	Flux graph^b^	Species	Reference	Condition	Flux graph
Total	118	581	581				
*Actinobacillus succinogenes*	2	5	5	*Mycobacterium tuberculosis*	1	5	5
*Agrobacterium tumefaciens*	1	8	8	*Penicillium chrysogenum*	1	2	2
*Arthrobacter sp.*	1	3	3	*Pichia pastoris*	3	7	7
*Ashbya gossypii*	1	2	2	*Pseudomonas aeruginosa*	1	2	2
*Aspergillus nidulans*	2	6	6	*Pseudomonasfluorescens*	1	1	1
*Aspergillus niger*	2	4	4	*Pseudomonas putida*	2	3	3
*Bacillus megaterium*	2	11	11	*Rhodobacter sphaeroides*	1	1	1
*Bacillus subtilis*	11	47	47	Rhodopseudomonas palustris	1	2	2
*Basfia Succiniciproducens*	1	1	1	*Saccharomyces cerevisiae*	14	76	76
*Chlorobaculum tepidum*	1	2	2	*Scheffersomyces stipitis*	1	4	4
*Corynebacterium glutamicum*	22	57	57	*Schizosaccharomyces pombe*	1	2	2
*Desulfovibrio vulgaris*	1	1	1	*Shewanella oneidensis*	1	3	3
*Escherichia coli*	32	297	297	*Shewanella spp.*	1	11	11
*Geobacillus thermoglucosidasius*	1	2	2	*Sinorhizobium meliloti*	1	1	1
*Geobacter metallireducens*	1	2	2	*Synechocystis sp.*	1	1	1
*Gluconacetobacter xylinus*	1	3	3	*Thermus thermophilus*	1	1	1
*Homo sapiens*	2	3	3	*Xanthomonas campestris*	1	1	1
*Methylobacterium extorquens AM1*	1	2	2	*Zymomonas mobilis*	1	3	3

^a^Condition stands for individual flux distribution that might be differentiated from the others by strain type, genotype and cultivation environment.

^b^Flux graph stands for the flux graph visualized by the CytoScape Web based on the corresponding flux values.

### Data Content

Each individual case of flux distribution includes several data fields describing the origin of the information, the process measure, a case-specific description and the flux distribution. The ‘Experiment name’ represents the citation information for the reference. The ‘Strain’ denotes the specific strain name of the organism if it was mentioned in the reference. The ‘Culture medium’ identifies the chemical composition of the growth medium. As the name suggests, the ‘Carbon source’ represents the carbon source in the medium. The ‘Growth rate’ indicates the average growth rate measure for cases that have a critical parameter determining the flux distribution. The ‘Specific rate’ includes specific uptake or secretion rates, available in the references, for carbon source and other primary metabolites with the unit of mmol g^−1^h^−1^. The ‘Case-specific description’ contains other information necessary for differentiating cases in a single experiment.

### Browse Function

**Figure 2.**
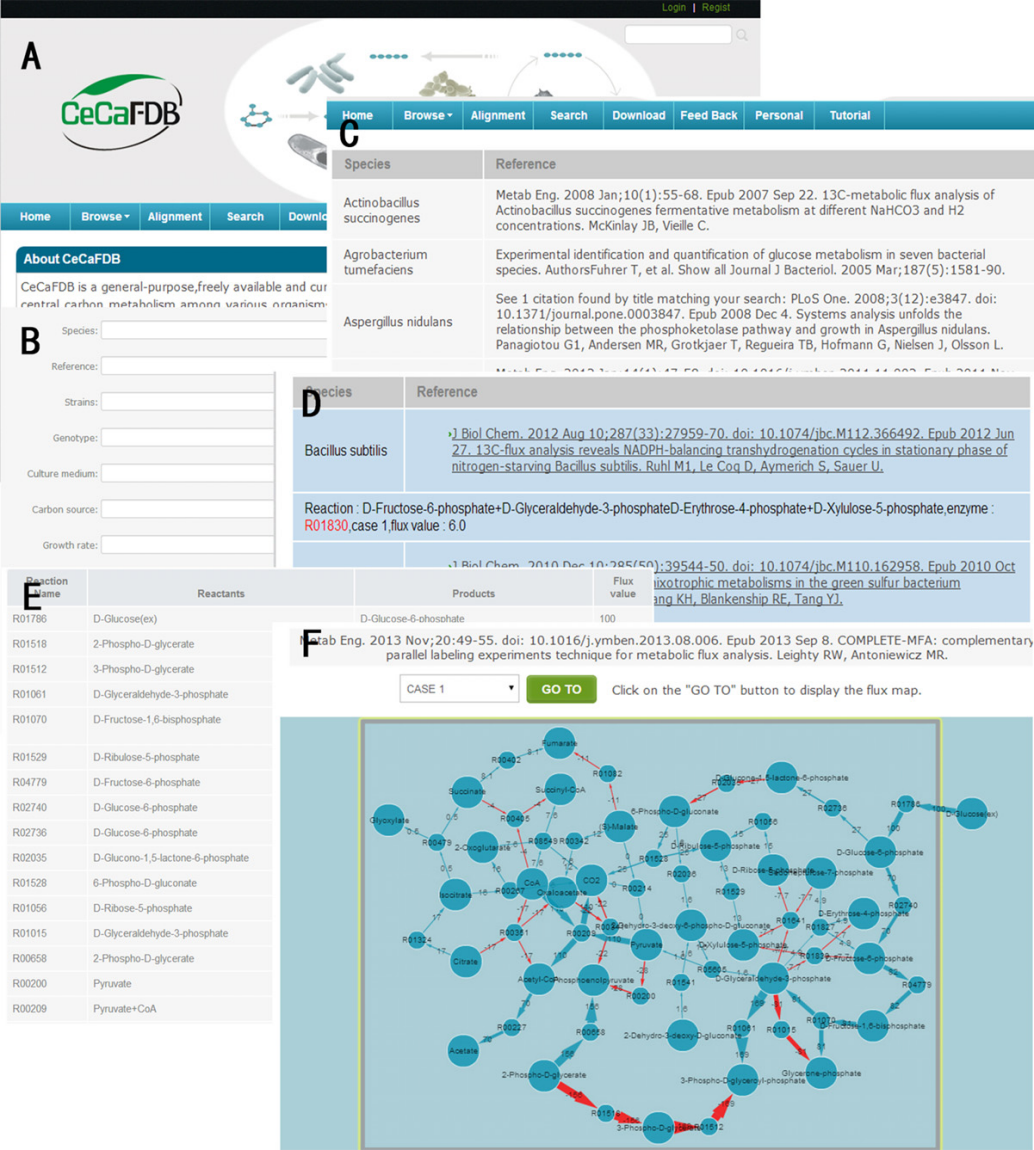
Screenshot montage of the CeCaFDB showing several of its data display and search tools. (**A**) Home page of CeCaFDB. (**B**) Flux distribution browser page. (**C**) Search panel. (**D**) Searching results page. (**E**) Example of flux distribution value. (**F**) Interactive graph representation of flux distribution of *Escherichia coli.*

Detailed information regarding where to locate and how to use the browsing tools is provided on the CeCaFDB website (Figure 2). The CeCaFDB provides two entry points for browsing items related to a complete description of a metabolic flux map.

### Browse metabolites and reactions

This entry point is directed to a page including the category information of the metabolites and reactions involved in all the flux maps. On this page, all the metabolites ‘belonging to the central carbon metabolism’ curated in the database were defined in a metabolite index list since the scope of CeCaFDB was concentrated on central carbon metabolism. Only reactions where the reactants and products belonged to the metabolite index list were included in the database. The reactions not meeting this description were excluded from our database due to the limited amount of literature. This web page contains a total of 66 kinds of metabolites and 76 kinds of chemical reactions constituting the central carbon metabolite systems for all of the collected organisms. For a more detailed introduction to the metabolites and reactions, the URLs were provided in the following column linking to the KEGG COMPOUND (36,37).

### Browse flux distribution

This entry is directed to the hierarchy and allows the users to taxonomically browse the database (Figure 2). Upon clicking the button, a summary page is displayed, providing a general description of the organisms and the relevant references. Clicking an organism name will display the whole list of references related to this organism. Users can easily navigate to the flux map pages within the reference (Figure 2). The structure of the flux map page contains several parts. The first part is an interactive graphical representation (see below) of the flux distribution based on the Flash-based Cytoscape Web software. A select box next to a ‘Go To’ button is located above the graph. To view the graph, you can select a favorite case of flux distribution through the select box and click the ‘Go To’ button.

The second part is a table representation of the flux distribution. Here, the ‘Reaction name’ denotes the KEGG code for a specific reaction. There are three types of ‘Reaction name’ in CeCaFDB. The first is a classical reaction code such as R01002 in KEGG, which denotes an elemental reaction in the central carbon metabolism. The second is labeled as ‘transport’ that represents all transport across biological membranes, such as malate transport or succinate transport. The third is labeled as ‘unknown’ that includes all lumped reactions connecting carbon sources to central carbon metabolites. The ‘Reaction’ displays the chemical equation that occurred in the reaction. The ‘Flux value’, the core of the database, displays the quantity of the flux value relative to the substrate uptake rate. In the case of multiple substrates, the sum of all the substrates uptake rates is set to 100.

The NADPH and ATP production capacities were recalculated based on the flux values. Where the flux values are missing for certain reactions contributing to NADPH or ATP production, the production rate is shown as N/A. The P/O ratio was set as 2.5 for NADH and 1.5 for FADH2. Since the substrate specificity of isocitrate dehydrogenase and malic enzyme might wobble between NADH and NADPH, a determined ATP or NADPH production rate is rarely available. Alternatively, CeCaFDB provides the maximum and minimum of NADPH and ATP production rates by assuming those enzymes operated exclusively with NADH or NADPH.

The third section of the page contains information for each flux distribution and a link to the original source of the reference. The description information contains subitems, including the ‘Strain’, ‘Culture medium’, ‘Carbon source’, ‘Growth rate’, ‘Specific rate’ and ‘Case-specific description’ (see above for descriptions of their meanings).

### Search Function

As with any web-enabled database, the CeCaFDB supports standard text queries. Users can combine multiple search terms in the easy search facility on the homepage, for example, ‘coli G6PDH’ will generate all of the studies that include the terms ‘coli’ and ‘G6PDH’. Furthermore, it is possible for users to further refine a search using the advanced search, which can be accessed using the SEARCH tab (Figure 2). In this functional module, the search facility offers access to search free text using the underlying data fields, including the experiment title, species name, genetic background, cultivation conditions and protocols, enzymes and flux values. For instance, if the ‘Enzyme’ field has ‘R00402’ (reaction number) entered and the ‘Flux value’ field has ‘45 and 50’ entered, the returned results will only contain flux distributions with enzyme R00402 and a flux value between 45 and 55. Figure 2D shows the resulting page when searching for a flux value between 45 and 55 across the entire CeCaFDB. Each hit contains two segments of information. The first is the species names and the second is the reference name and the matching field, which is emphasized by red color in the search. Users can simply click on the reference title to learn more of the details.

### Interactive visualization of flux distribution

The CeCaFDB utilizes the Cytoscape Web API to visualize representations of the metabolic flux maps (35). This interactive visualization tool has its roots in the popular Cytoscape Web platform, but uses Flash technology instead of Java to reduce launch time. It is compatible with any web browser. The latest version of Cytoscape Web works best with up to several thousands of nodes and edges that completely satisfies the requirements of flux map visualization. The flux map is delivered into the Cytoscape Web API using selected parameters, drawing a dynamic graphical display that enables users to move and modify the node and edge properties. Furthermore, the graph can be panned and zoomed in the same layout. In the default configuration, a metabolite is denoted by a blue circle and the reaction by a blue eclipse (Figure 2). A light blue edge denotes a forward flux, whereas a light red edge represents a backward reaction. The edge width is proportionate to the flux value, which is also displayed on the graph with black letter. The boundary between the compartmentation of the organelle and the intra/extracellular environment is represented by closed brown lines, and the compartmentation names are displayed. The chemical transport between different compartmentations and the substrate uptake are embodied by the layout to ensure a meaningful picture.

### Flux Comparison Function

#### Vector-based comparison

An intuitive representation of the flux distribution takes the form of a vector *V* whose element is the value of each reaction in the concerned metabolic network (12). In such a way, the comparison and analysis between the flux distributions can be accessed by the angular cosine of different distribution vectors

(1) \begin{equation*} {\rm Similarity} = \frac{{\mathop \sum \nolimits_{i = 1}^n A_i \times B_i }}{{\sqrt {\mathop \sum \nolimits_{i = 1}^n \left( {A_i } \right)^2 } \sqrt {\mathop \sum \nolimits_{i = 1}^n \left( {B_i } \right)^2 } }}\end{equation*}

*A* and *B* represent different vectors and *n* represents the dimension of the vectors.

#### Stoichiometry-based comparison

The vector treatment of the flux distribution comparison takes different reactions into equal consideration without losing information regarding the relative weight of the reactions. However, the reaction's impact on the material flow of the metabolic network varies based on the diverse number of consumed reactants and different amounts of product, namely the stoichiometric constraints. Hence, a more appropriate representation of the flux distribution and a description of the flux comparison should incorporate the chemical stoichiometric constraints into the flux value. With the intention of describing the flux distribution with stoichiometric information, the flux matrix *F* was created by multiplying the stoichiometric matrix by the flux vector as follows:

(2) \begin{equation*} F = S'V' \end{equation*}

where *S* stands for the stoichiometric matrix of a metabolic network. The flux matrix *F* incorporates the chemical stoichiometric constraints into the description of the flux value and is suitable for further comparison of the flux distribution. In the flux matrix framework, similarity between the flux distributions can therefore be defined as follows:

(3) \begin{equation*} {\rm Similarity} = \frac{{AB'}}{{\sqrt {\mathop \sum \nolimits_{i,j = 1}^{l,m} \left( {A_{ij} } \right)^2 } \sqrt {\mathop \sum \nolimits_{i,j = 1}^{l,m} \left( {B_{ij} } \right)^2 } }}\end{equation*}

where *A* and *B* stand for the different flux matrixes. *l* and *m* are the dimensions of the matrixes. This similarity measurement has assimilated information regarding both the flux values and stoichiometric coefficients; therefore, it might be a better approximation of the similarity of the flux distribution.

#### Topology-based comparison

However, even the stoichiometry-based method cannot represent solid consideration of a metabolic flux comparison. The metabolic flux is flowing through a metabolic network with a specific topological structure for a particular function. Because the topology and function relationship is of great importance for all the metabolic networks (38), it is necessary for the comparison tool to be powerful enough to take into account both the network architecture similarities and the node similarities (39,40). To achieve this objective, a pathway alignment algorithm was modified from the one proposed by Zhengping *et al*. (40) and was combined with the flux value weight to adapt to flux distribution comparisons. In short, the metabolic flux distribution comparison problem was based on a weighted directed graph alignment representation. The similarity calculation between the two metabolic flux maps was then transformed into an optimal weighted alignment between the two graphs.

A metabolic flux distribution with *l* metabolites and *n* reactions (see example in Supplementary Figure S2) can be treated as a weighted directed graph *G(N,E,W)* whose nodes *N* correspond to the reactions and whose edges *E* connect with the nodes if the product of one reaction serves as the substrate of the other (40). Before formulating the flux comparison problem with a graphical representation, we should first parameterize the weighted adjacent matrix and node-similarity matrix.

#### Weighted adjacent matrix

Let *m_ij_* denote the summed stoichiometry coefficients of the compounds shared by the products of the *i-*th reaction and the reactants of the *j*-th reaction. The $m_{ij} = \delta \left( {\left| {\sum\limits_{ki} {S_{ki} } } \right| + \left| {\sum\limits_{kj} {S_{kj} } } \right|} \right)$. *δ* equals 1 when $\mathop \sum \limits_{ki} s_{ki}\geq 0$ and *δ* equals −1, when $\sum\limits_{ki} {s_{ki}< 0}$. The value of *m_ij_* is set to zero when no common compounds are shared by the two reactions. Let *v_i_* denote the flux value of the *i*-th reaction. The weight of the graph is introduced by constructing the weighted adjacent matrix *W* {*w_ij_*}, which is elaborately designed to incorporate both the flux value and stoichiometry. The determination of the elements of *W* is described by Equation (4) and a simplified example is displayed in Supplementary Figure S2.

(4) \begin{equation*} w_{ij} = \left\{ {\begin{array}{*{20}l} {\frac{{v_i }}{{\sqrt {\mathop \sum \nolimits_{i = 1}^n \left( {v_i } \right)^2 } }},i = j} \\ {\frac{{m_{ij} }}{{\sqrt {\mathop \sum \nolimits_{i,j}^n \left( {m_{ij} } \right)^2 } }} = \frac{{ - m_{ji} }}{{\sqrt {\mathop \sum \nolimits_{i,j}^n \left( {m_{ij} } \right)^2 } }},i \ne j} \\ \end{array}} \right. \end{equation*}

### Node similarity function

There have been several similarity functions proposed to produce a similarity score between a pair of reactions or enzymes, such as functions based on the similarity of amino acid sequences or information content regarding an enzyme class hierarchy (41–43). In our model, a function was based on the probability that two enzymes are the same in the enzyme hierarchy. The enzyme hierarchy is the hierarchy constructed with the EC numbering system (e.g. [3:2:2:1], [3:2:1]). For the two enzymes *u* and *v*, a common upper class is defined as the enzyme class *h_uv_*, which is the lowest class in the upper classes of enzymes on the enzyme hierarchy. For the same enzymes, their common upper class is their enzyme class. For example, [2:2:3] is the common upper class between [2:2:3:4] and [2:2:3:5]. The *C* (*h*) expresses the number of elements for all of the enzymes whose classes are included under the enzyme class *h*. A similarity function between *e_u_* and *e_v_* is defined as follows:

(5) \begin{equation*} S_{uv} = \frac{1}{{C\left( {h_{uv} } \right)}}\end{equation*}

which provides a normalized similarity function that incorporates values in the interval [0, 1].

In our algorithm, given two metabolic flux maps *G*_1_ = *G*_1_ (*N*_1_, *E*_1_, *W*_1_) and *G*_2_ = *G*_2_ (*N*_2_, *E*_2_, *W_2_*), where $N_1 = \{ n_1^1 ,n_2^1 , \ldots ,n_o^1 \}$ and $N_2 = \{ n_1^2 ,n_2^2 , \ldots ,n_p^2 \}$, the weighted adjacent matrixes of *G*_1_ and *G*_2_ are *A* = (*a**_ij_***)*_oxo_* and *B* = (*b**_ij_***)*_pxp_*, as constructed in Equation (5). As suggested by Zhengping *et al*. (40), the matching between nodes $(n_i^1 ,n_k^1 ) \in N_1$ and $(n_j^1 ,n_l^1 ) \in N_1$ and between edges $(n_i^1 ,n_k^1 ) \in N_1$ and $(n_j^1 ,n_l^1 ) \in N_1$ is represented by the binary variables *x_ij_* and *y_ijkl_* respectively, as follows:

(6) \begin{equation*} \begin{array}{l} x_{ij} = \left\{ {\begin{array}{*{20}c} {{\rm one}, {\rm if}\,\, n_{i}^1 \in N_{1}\,\, {\rm matches}\,\,n_{j}^2 \in N_2 } \\ {{\rm zero},{\rm otherwise}} \\ \end{array}} \right. \\ \\ y_{ijkl} = \nonumber \left\{ {\begin{array}{*{20}c} {{\rm one},\, {\rm if}\,\, n_{j}^1 \in N_1 \,\,{\rm matches}\,\, n_{j}^2 \in N_2\,\, {\rm and}\,\, n_k^1 \in N_1 \,\, {\rm matches}\,\, n_{j}^2 \in N_2 } \\ {{\rm zero},{\rm otherwise}} \\ \end{array}} \right. \\ \end{array} \end{equation*}

Obviously, each *X* = {*x_ij_*} and *Y* = {*y_ijkl_*} determine the local alignment between two flux maps *G*_1_ and *G*_2_. The similarity between the two flux maps *G*_1_ and *G*_2_ according to a given alignment matrix *X* of nodes, is thus calculated as a sum score including both the node and edge matching scores in an objective function, as in Equation (7), which is similar to the form of the inner product of the flux vector.

}{}{\begin{equation*} max_X f\left( {G_{1,} G_2 } \right) = {\rm \lambda }\mathop \sum \limits_{i = 1}^o \mathop \sum \limits_{j = 1}^p S_{ij} a_{ii} b_{jj} x_{ij} + \left( {1 - {\rm \lambda }} \right)\mathop \sum \limits_{i = 1}^o \mathop \sum \limits_{j = 1}^p \mathop \sum \limits_{k = 1}^o \mathop \sum \limits_{l = 1}^p a_{ik} b_{jl} y_{ijkl} \end{equation*}

(7) \begin{equation*} s.t.\left\{ \begin{array}{l} \mathop \sum \limits_{j = 1}^p x_{ij} \le 1,i = 1,2, \ldots ,o \\ \mathop \sum \limits_{i = 1}^o x_{ij} \le 1,j = 1,2, \ldots ,p \\ x_{ij} \ge y_{ijkl} ,i,k = 1,2, \ldots ,o;j,l = 1,2, \ldots ,p \\ x_{kl} \ge y_{ijkl} ,i,k = 1,2, \ldots ,o;j,l = 1,2, \ldots ,p \\ x_{ij} = 0/1,i,k = 1,2,...,o;j,l = 1,2,...,p \\ y_{ijkl} = 0/1,i,k = 1,2, \ldots ,o;j,l = 1,2, \ldots ,p \\ \end{array} \right. \end{equation*}

The first two constraints ensure that the relationship between two nodes is a one-to-one correspondence or involves no matches. The third constraint implies the integer constraint for the variable *x*. In this framework *λ* is a scaled parameter between 0 and 1 that is aimed at reaching a compromise between the node (flux value) and edge (stoichiometry) score. The structure of this objective function provides a normalized similarity function that incorporates values from the interval [−1, 1]. A pair of identical flux maps yield a similarity function of 1, whereas a pair of flux maps with identical structures and reversed reactions yield a similarity function of −1.

### Alignment tab

Based upon the three algorithms mentioned above, the CeCaFDB's Alignment Tab provides four different comparison tasks: ‘Vector-based similarity’, ‘Stoichiometry-based comparison’, ‘Enzyme Topology-based similarity’ and ‘Topology-based similarity’. For the vector- and stoichiometry-based tasks, a calculation is carried out on the shared reaction set between the two flux distributions. The ‘Enzyme topology-based similarity’ is implemented using integer programming by taking *λ* as 1. The ‘Topology-based similarity’ should be launched with a user-designated parameter-vertex to the edge score balance (*λ* value), which determines the relative impact of the vertex and edge scores on the final similarity score. This can be fine-tuned according to the user's need. We have implemented a proposed integer-programming algorithm using the YALMIP and Gurobi softwares. The 2D X and 4D Y were transformed into 1D data and compressed into one single array to save memory requirements and to adapt to the requirement of the optimization package.

By clicking the ALIGNMENT tab, user will enter the flux comparison page. On this page, there are three select boxes for ‘species’, ‘reference’ and ‘flux distribution’. Through the use of these three boxes, the specific flux distributions to be compared can be selected by the user. After that, clicking the SELCET button will transfer the selected case to the SELECTED box. Repeating this process will add all the desired cases to the SELCETED box. The cases can be deselected by clicking the REMOVE button. Clicking the COMPARISON button will initiate a pairwise similarity analysis on the flux distributions in the SELECTED box except that the ‘Topology-based similarity’ currently receives the alignment job only between two flux distributions.

The comparison results from the latter two methods contain a similarity score, a *P-*value and an URL link to the graphical representation of the alignment solution. The statistical *P*-value was calculated with a Monte Carlo permutation test, in which the same comparison was executed against a 100-flux map with random sampled values and with the *P-*value obtained by counting the fraction of the flux distributions containing alignments that received higher scores (40). The legends in the graphical representation of the flux comparisons are similar to those in the flux visualization, except that the olive line connecting the enzymes represents the relationship match between the two flux maps, whereas the line width was proportionate to the contribution factor from each enzyme pair to the ultimate similarity score. In addition, on this graphical representation page, CeCaFDB offers a downloadable txt file with tabular form information about the matched reactions, conserved metabolic pathways, gaps in the network and solely inserted reactions. Relying on this alignment tab, CeCaFDB supports flux map query tasks against all the curated flux map data through similarity comparisons after the data have been submitted to CeCaFDB.

### Data Download and Submission

Users may download the CeCaFDB data containing metabolic networks and flux distribution vectors. Upon clicking the Download tab, a user will open a download page. Clicking the corresponding reference name will display the links for the corresponding flux distribution that is stored in an Excel file.

The CeCaFDB receives submissions of the flux distribution data. The submission of data requires an account within the CeCaFDB, which can be obtained through online registration. For submissions, the flux data should be formatted in a template file on the submission page, which is similar to the input template for the VANTED software (44). The input file should contain the chemical equations and E.C numbers (or KEGG reaction number) of the metabolic network and the flux distribution values. Particularly important are the lumped reactions of the uploaded data, which should be broken down into their original forms as in the KEGG to be consistent with other data.

## COMMON USE CASES

The combination of data available from the CeCaFDB and the comparison functions via the web interface offer a powerful tool for structural comparisons and functional discovery of metabolic flux distribution. A few examples are provided below.

### Flux Map Comparison

**Figure 3.**
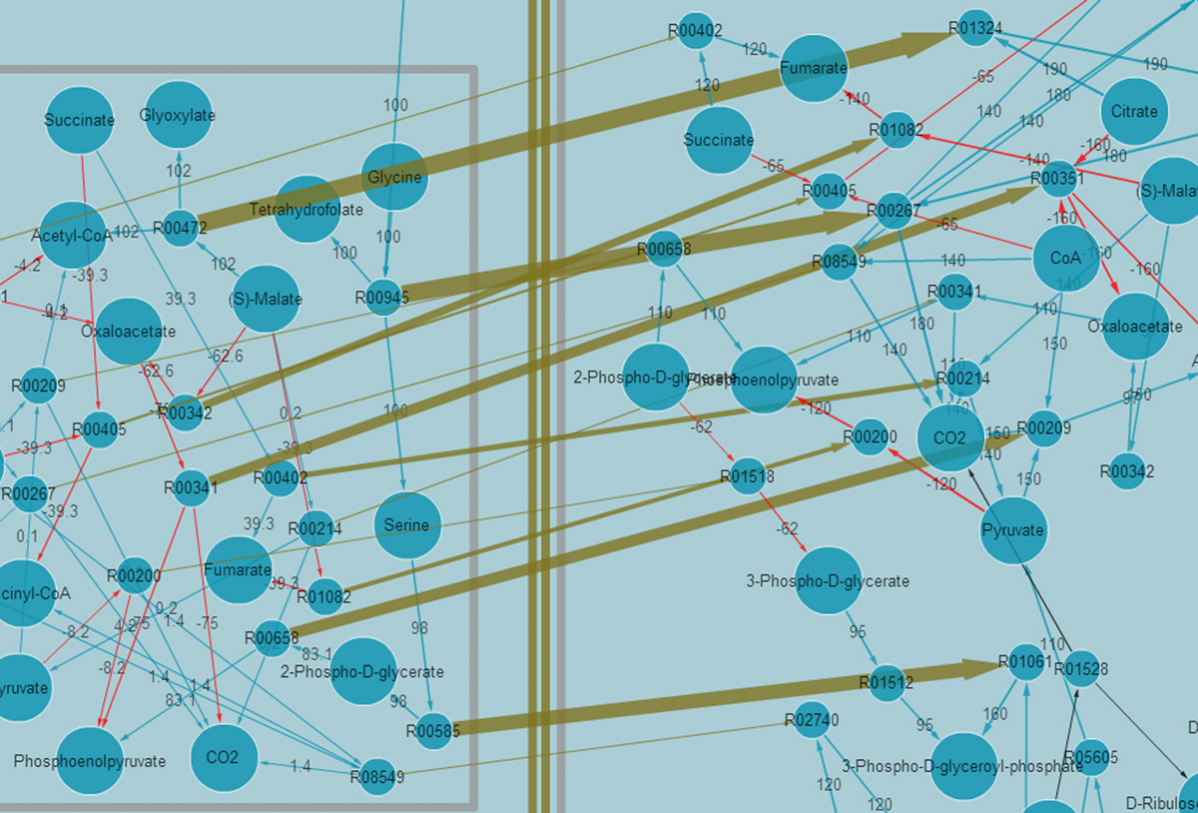
Example of an alignment between the flux distributions of *M. extorquens* AM1 and *E. coli*. The submap containing the enzymes L-Malate glyoxylate-lyase (R00472), glycine hydroxymethyltransferase (R00945), phosphoenolpyruvate carboxykinase (R00341) and malate dehydrogenase (R00342) in *M. extorquens* AM1 matches best with the submap containing the enzymes aconitase (R01324), isocitrate dehydrogenase (R00267), citrate synthase (R00351) and fumarate hydratase (R01082) in *E. coli*. Another part of the *M. extorquens* AM1 corresponding to enolase (R00658) and transaminase (R00585) matches well with that of pyruvate oxidation (R00209) and glyceraldehyde 3-phosphate dehydrogenase (R01061) in *E. coli*. The other elements of *M. extorquens* possess little consensus with *E. coli*.

Clicking the alignment tab and selecting the flux distribution of *Methylobacterium extorquens* AM1 under mineral salts medium with methanol as the carbon source (45) and *Escherichia coli* under M9 minimal medium with glucose as the carbon source (46), and then performing an alignment based upon the ‘Topology-based similarity’ algorithm, produces a comparison result between the two flux distributions. The result includes a similarity score, a *P-*value and a URL linking to the graphical representation of the alignment solution. The alignment graph is directly displayed with a Cytoscape Web API (Figure 3). The olive line connecting the enzymes represents the matching relationship between the two flux maps. From Figure 3, it can be seen that the submap containing the enzymes L-Malate glyoxylate-lyase (R00472), glycine hydroxymethyltransferase (R00945), phosphoenolpyruvate carboxykinase (R00341) and malate dehydrogenase (R00342) in *M. extorquens* AM1 is the best match with the submap containing the enzymes aconitase (R01324), isocitrate dehydrogenase (R00267), citrate synthase (R00351) and fumarate hydratase (R01082) in *E. coli*. Another part of the *M. extorquens* AM1 corresponding to enolase (R00658) and transaminase (R00585) matches well with that of pyruvate oxidation (R00209) and glyceraldehyde 3-phosphate dehydrogenase (R01061) in *E. coli*. The other elements of *M. extorquens* possess little consensus with *E. coli*.

### Interrelation between Flux Distributions

A key feature of the CeCaFDB is its extensive support for flux distribution queries against the selected part of the database, providing valuable hints regarding the interrelationships between them. Once the submitted flux distributions have been approved by administrators, users can perform similarity calculations on the submitted data using other data and can draw interrelations between all of the selected data. As an example, we conducted a correlation analysis on the flux distributions of *E. coli* under genetic and environmental manipulation. The correlation was analyzed for a total of 18 flux distributions from three references, with 10 of them being knocked out in different transcriptional factors or enzymes and 10 of them being cultivated under cultivated mediums and carbon sources (47–49). The detailed information for the flux distribution is shown in Supplementary Table S1. Supplementary Figure S2 indicates the hierarchy cluster tree of the ultimate results. Within the cluster tree, the FhlA and FadR mutants were located in the center and those under acetate or high glucose circumstances were isolated from the other cases. In the clustering process, the genetically and environmentally modified cases were intermixed. According to the cluster tree, we discovered that the distribution of the genetically modified flux is sparser than that from the changed environments, which points to a more stringent environmental effect on the flux distribution compared with the genetic manipulation on this occasion.

## SUPPLEMENTARY DATA

Supplementary Data are available at NAR Online.
